# Prognostic Factors for Long-Term Survival in Patients with Ampullary Carcinoma: The Results of a 15-Year Observation Period after Pancreaticoduodenectomy

**DOI:** 10.1155/2014/970234

**Published:** 2014-03-02

**Authors:** Fritz Klein, Dietmar Jacob, Marcus Bahra, Uwe Pelzer, Gero Puhl, Alexander Krannich, Andreas Andreou, Safak Gül, Olaf Guckelberger

**Affiliations:** ^1^Department of General, Visceral, and Transplantation Surgery, Charité Campus Virchow Universitätsmedizin Berlin, 13353 Berlin, Germany; ^2^Department of General and Visceral Surgery, Bielefeld Evangelical Hospital, 33617 Bielefeld, Germany; ^3^Department of Hematology/Oncology, Comprehensive Cancer Center, Charité Universitätsmedizin Berlin, 13353 Berlin, Germany; ^4^Department of Biostatistics, Coordination Center for Clinical Trials, Charité Universitätsmedizin Berlin, 13353 Berlin, Germany

## Abstract

*Introduction*. Although ampullary carcinoma has the best prognosis among all periampullary carcinomas, its long-term survival remains low. Prognostic factors are only available for a period of 10 years after pancreaticoduodenectomy. The aim of this retrospective study was to identify factors that influence the long-term patient survival over a 15-year observation period. *Methods*. From 1992 to 2007, 143 patients with ampullary carcinoma underwent pancreatic resection. 86 patients underwent pylorus-preserving pancreaticoduodenectomy (60%) and 57 patients underwent standard Kausch-Whipple pancreaticoduodenectomy (40%). *Results*. The overall 1-, 5-, 10-, and 15-year survival rates were 79%, 40%, 24%, and 10%, respectively. Within a mean observation period of 30 (0–205) months, 100 (69%) patients died. Survival analysis showed that positive lymph node involvement (P = 0.001), lymphatic vessel invasion (P = 0.0001), intraoperative administration of packed red blood cells (P = 0.03), an elevated CA 19-9 (P = 0.03), jaundice (P = 0.04), and an impaired patient condition (P = 0.01) are strong negative predictors for a reduced patient survival. *Conclusions*. Patients with ampullary carcinoma have distinctly better long-term survival than patients with pancreatic adenocarcinoma. Long-term survival depends strongly on lymphatic nodal and vessel involvement. Moreover, a preoperative elevated CA 19-9 proved to be a significant prognostic factor. Adjuvant therapy may be essential in patients with this risk constellation.

## 1. Introduction

Ampullary carcinomas arise from the ampulla or papilla of Vater (the duodenal papilla) and account for 0.2% of tumors of the gastrointestinal tract. However, with a proportion of 7% to 9%, they represent the second largest proportion (after pancreatic carcinoma) of periampullary carcinomas, which include ampullary carcinomas and carcinomas of the pancreas, the distal bile duct, and the periampullary duodenum [1–3]. In contrast with other carcinomas of the periampullary region, ampullary carcinomas have a higher resection rate, a lower recurrence rate, and a better overall prognosis [3–6]. To date, the etiology of ampullary carcinoma has not been clearly identified. An adenoma-to-carcinoma sequence similar to that of colon carcinoma has been described for ampullary carcinoma [7].

In traditional terms, ampullary cancer is already distinguished from carcinomas of the pancreas, bile duct, and duodenum. For one thing, due to their anatomical location, ampullary tumors become clinically apparent early because of bile or pancreatic duct occlusion [8]. Thus, ampullary carcinomas are often diagnosed at an early tumor stage and, therefore, have a higher probability of successful surgical resection [4]. Secondly, the 5-year survival rate is reported with up to 39%, which is between that of duodenal carcinoma (59%) and carcinomas of the pancreas or bile duct (15% and 27%, resp.) [3, 9, 10]. A reason for the better overall prognosis may be the difference in the histological origin of ampullary carcinomas. As early as 1963, Whipple reported that ampullary cancers are more likely to be of the adenomatous type with less general lymphatic and blood vessel invasion [11]. Current histopathological studies have also suggested further subdivision of ampullary carcinomas based on their exact histopathological findings [12, 13]. For example, intestinal ampullary adenocarcinomas arise from the surrounding intestinal epithelial layer, whereas pancreatobiliary ampullary cancers originate in the endothelium of the distal bile duct or pancreatic duct [14, 15].

Computed tomography (CT) and magnetic resonance imaging cholangiopancreatography (MRCP) constitute the current clinical diagnostic methods of choice. Other methods, such as endosonography and endoscopic retrograde cholangiopancreatography (ERCP), allow for sample collection and thus permit further histological differentiation. A radical pancreaticoduodenectomy, performed either as a pylorus-preserving pancreatic head resection (PPPD) or a classic Whipple procedure (KW), is considered to be the gold standard therapy for ampullary carcinoma. Currently, endoscopic papillectomy is increasingly performed as an initial intervention in suspected benign papillary tumors [16, 17]. The decision to perform a subsequent pancreaticoduodenectomy may be based on the histopathological finding of the resected specimen. The resectability of ampullary carcinoma with a curative intention is 76.5% to 89.4% [2, 18]. Due to the rarity of this tumor, studies describing the long-term progress are scarce and are available only for up to 10 years after resection. Overall long-term survival still remains low. A major component in this issue is tumor recurrence. The aim of this retrospective study was to identify factors that influence the long-term survival in a large patient population over 15 years.

## 2. Patients and Methods

### 2.1. Preoperative Data

Between 1992 and 2007, 143 patients underwent resection of histologically verified ampullary cancer at our institution. Of these patients, 87 (61%) were men and 56 (39%) were women, with a median age of 64 (33–83) years. The median body mass index (BMI) in the patient group was 24.8 (13.5–38.8) kg/m^2^. Forty-three (30%) patients presented with a Karnofsky index below 80%. Nicotine consumption was noted in 44 (31%) patients and regular alcohol consumption in 32 (22%) patients. Preoperative symptoms were apparent in 130 (91%) patients. Seventy-five (52%) patients presented with jaundice, and 88 (62%) patients had nonspecific epigastric pain. Twenty-nine (20%) patients described a weight loss of more than 10 kg in the three months preceding the presentation. Permanent nausea affected 29 (20%) patients, and a reduced performance status was experienced by 27 (19%) patients (Table 1). Twenty-one (15%) patients already presented with diabetes mellitus, of whom 15 (10%) were insulin-dependent and 6 (4%) were on oral antidiabetics. Thirteen (9%) patients had a history of pancreatitis. In the context of diagnosis, 121 (85%) patients had an abdominal CT, and a tumor was diagnosed in 56 cases (46% of all CT examinations). An endosonography was performed in 34 (24%) patients, with tumor findings in 22 (65% of all endosonographies) patients. Preoperative endoscopic retrograde cholangiography (ERC) was performed in 131 patients (92%), with evidence of tumor in 105 patients (73%). A papillotomy was undertaken in 57 patients (40%), and preoperative stent placement in the common bile duct was performed in 39 patients (27%). Preoperative laboratory chemical examinations gave a median CA 19-9 value of 23 U/L (1–9171), a bilirubin level of 1.7 mg/dL (0.2–44.4), and a *γ*GT of 172 U/L (6–1865).

### 2.2. Surgical Procedure

In 86 (60%) patients a pylorus-preserving pancreaticoduodenectomy (PPPD) was performed and in 57 (40%) patients a Kausch-Whipple pancreaticoduodenectomy (KW) was performed. Pancreatoenteral anastomosis was performed as pancreaticojejunostomy in 123 patients (86%) or pancreaticogastrostomy in 20 patients (14%) using a mattress suture technique in 98 patients (69%) and Cattell duct-to-mucosa technique in 45 patients (31%). Due to tumor infiltration, partial portal vein resection has been performed in two (1%) patients. Reconstruction of the superior mesenteric artery was indicated in one patient (1%). The median operation time was 325 (182–785) minutes, with an average blood loss of 500 mL (100–3000). A total of 43 (30%) patients were intraoperatively substituted with packed red blood cells (PRBC). Pancreatic reconstruction was performed in 132 (92%) patients as pancreaticojejunostomy (PJ) and in 11 patients as pancreaticogastrostomy (PG) (8%). The operation was extended in nine (6%) patients, with four patients receiving a partial liver resection, two a splenectomy and partial colon resection, and one a nephrectomy. Intraoperative complications occurred in 4 (3%) patients; three patients had bleeding that was difficult to control and one patient experienced both myocardial infarction and cardiac arrhythmia. All of the operations were performed in line with tumor-surgical criteria by experienced visceral surgeons who were taking a curative approach. Both PPPD and KW were performed in accordance with international standards as en bloc dissection with lymphadenectomy along the hepatoduodenal ligament, celiac trunk, and superior mesenteric artery. The resection areas were classified intraoperatively as curative (R0) when no microscopic evidence of tumor cells was present histopathologically. The tumor stage was graded using the UICC classification of 2009 for ampullary cancers [19].

### 2.3. Standard Postoperative Care

Every patient received a nasogastric tube for gastric decompression. Amylase and/or lipase levels were monitored daily in the serum and in the intraoperatively placed abdominal drains (Degania Silicone Europe GmbH, Regensburg, Germany) on the first and fourth postoperative days. Radiological contrast imaging was performed on the fifth postoperative day over the nasogastric tube.

The diagnosis of a postoperative pancreatic fistula formation (POPF) was based on the definition of the International Study Group on Pancreatic Fistula (ISGPF) [20]. The levels of amylase in the intraoperatively placed drains were not available for all subjects in our database. The lipase levels in the drains had always been measured. We therefore slightly modified the ISGPF definitions and used amylase or lipase levels in the drains to define the existence of a POPF. Postpancreatectomy hemorrhage (PPH) and delayed gastric emptying (DGE) were also defined based on the International Study Group of Pancreatic Surgery (ISGPS) definitions. [21, 22]. However, the definitions of ISGPS for POPF, PPH, and DGE were not published until 2004 and 2007, respectively. Thus, incidences of POPF and PPH had to be retrospectively evaluated.

### 2.4. Statistics

The data were collected in a database (Microsoft Access 2.0, Microsoft Corporation, Seattle, USA) and evaluated retrospectively. Unless otherwise specified, the data are expressed as median and range. Survival analysis was determined by means of the Kaplan-Meier method (log-rank test) and specific risk factors by the Mann-Whitney *U* test using SPSS for Windows 14.0 (SPSS Inc. Chicago, IL, USA). A *P* value below 0.05 was considered to be significant.

## 3. Results

### 3.1. Postoperative Progress and Surgical Complications

The median length of hospital stay was 16 (9–100) days. The median stay in intensive care was 3 (1–74) days. Twelve (8%) patients developed POPF requiring operative revision in five cases. Insufficiency of the bile duct anastomosis occurred in two (1%) patients. In total, revision surgery was undertaken in 10 (7%) patients (Table 2). These revisions comprised four residual pancreatectomies and one new installation of the pancreatoenteral anastomosis (a pancreaticogastrostomy was followed by a pancreaticojejunostomy) and three revisions for wound dehiscence and two instances of PPH. Postoperative delayed gastric emptying occurred in 8 patients (6%). The perioperative lethality was 3.5%. The cause of death was sepsis in two patients, and one patient had surgically untreatable bleeding, cardiac decompensation from known cardiac insufficiency, or acute myocardial infarction. Within the observation period, 18 (13%) patients underwent in-patient readmission. Of these, 10 (7%) patients were operated on again for reasons unrelated to the underlying condition. Emerging diabetes mellitus was diagnosed in 9 (6%) patients, and 64 (45%) patients needed postoperative enzyme substitution at mealtimes. The total mortality was 69% in a median postoperative observation period of 30 months (0–205).

### 3.2. TNM

The histological examination of the pathological specimen and categorization by means of TNM classification resulted in a pTis stage in 2 (1%) patients and a pT1 stage in 14 (10%) patients. An almost identical number of patients had pT2 (53 patients; 37%) and pT3 stages (54 patients; 38%). In 20 (14%) patients a pT4 stage was diagnosed. Positive lymph node involvement (pN1) was evident in 69 (48%) cases. More than half of the patients were in a G2 stage (75 patients; 52%) of differentiation (pG), followed by stage G3 in 36% (52 patients). Fifteen (10%) patients presented with a G1 stage and 1 (1%) patient with a G4 stage. The tumor size was smaller than 2 cm in diameter in 53 patients (37%) and bigger than 2 cm in 90 patients (63%).

Microscopically detected tumor infiltration, detectable by microscopy (R1) of the resection margins or at the retropancreatic ablation level, was evidenced in 12 (8%) patients. Lymphatic invasion was present in 70 (49%) patients and vascular invasion in 17 (12%) patients in the final histology.

### 3.3. UICC Stages

As a result of classifying the 143 patients as per the UICC stages, 16 (11%) patients were stage 1a and 33 (23%) patients were stage 1b. Stage 2b, with 51 patients (35%), was the most frequent. In comparison, 20 patients (14%) were stage 2a and 16 (11%) and 7 (6%) patients were stages 3 and 4, respectively.

### 3.4. Survival and Prognostic Factors

**Figure 1:**
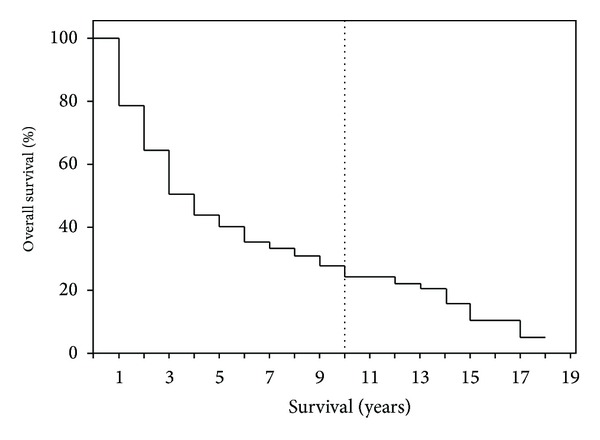
The overall survival for patients after the resection of ampullary carcinoma with curative intention.

**Figure 2:**
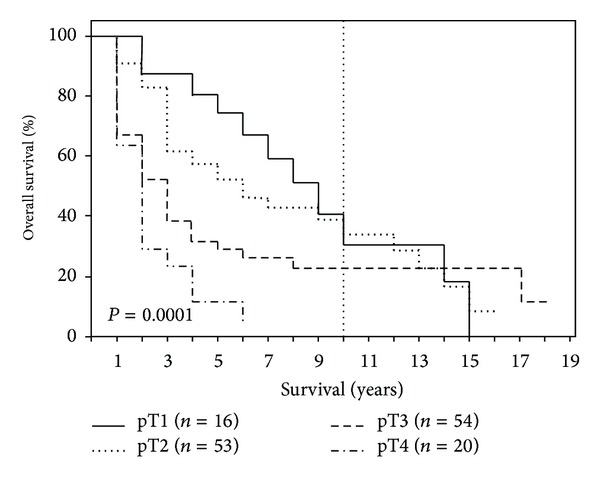
Survival depending on tumor stage (pT1, pT2, pT3, and pT4).

**Figure 3:**
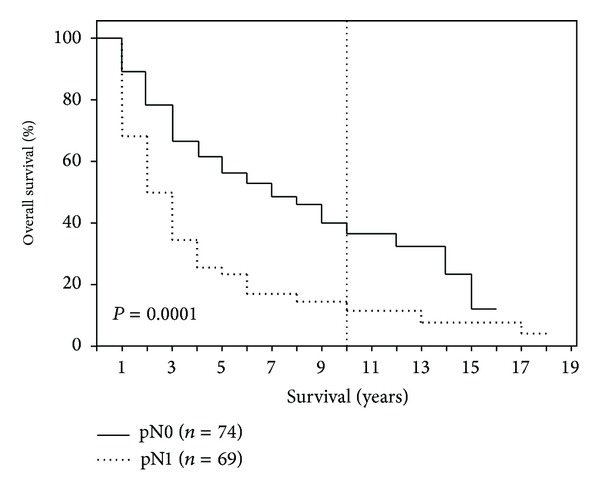
Survival according to lymph node status (pN0 versus pN1).

**Figure 4:**
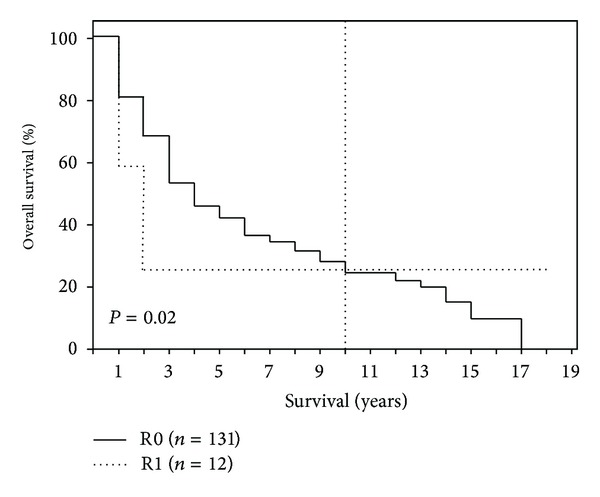
Survival depending on surgical radicality (R0 versus R1).

**Figure 5:**
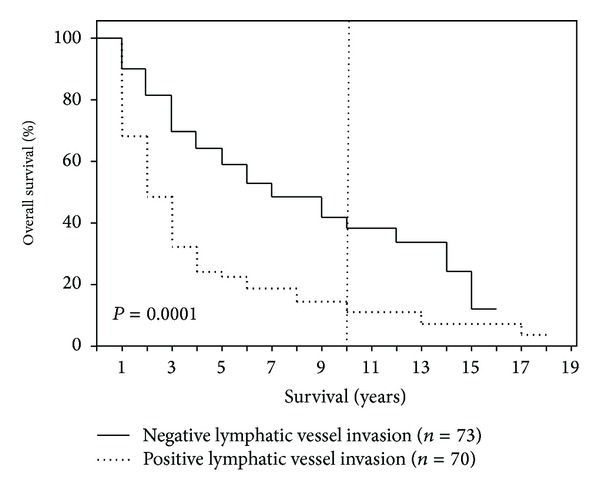
Survival depending on lymphatic vessel invasion (L0 versus L1).

After 1-, 5-, 10-, and 15-year periods, the overall survival of the examined patient population was 79%, 40%, 25%, and 10%, respectively, with a median survival term of 37 months (Figure 1). Survival analysis (log-rank) resulted in a significantly reduced survival for patients who had a reduced general condition (*P* = 0.008), required intraoperative administration of PRBC (*P* = 0.003), had POPF (*P* = 0.013), had an advanced tumor stage (*P* = 0.0001) (Figure 2), had a pT4 tumor invasion depth (*P* = 0.0001), had a positive lymph node stage (0.0001) (Figure 3), had a pG4 tumor grade (*P* = 0.0001), had a microscopically or macroscopically positive resection margin (*P* = 0.02) (Figure 4), had vascular (*P* = 0.008) or lymphatic invasion (*P* = 0.0001) (Figure 5), and had a preoperatively elevated CA 19-9 (*P* = 0.008). There were no significant differences in regard of overall survival in patients who received a PPPD and patients who underwent classic Whipple procedure (*P* = 0.222). A tumor size smaller than 2 cm did not have a significant effect on overall survival (*P* = 0.458). Examining the risk factors with respect to survival, multivariate analysis revealed that the following are risk factors for poor prognosis: lymphatic invasion (*P* = 0.000), intraoperative administration of PRBC (*P* = 0.008), and a preoperatively elevated CA 19-9 (*P* = 0.023) (Table 3).

## 4. Discussion

In 1912, Hirschel conducted the first documented single-stage resection of an ampullary carcinoma in Heidelberg, Germany [23]. Since then, morbidity and mortality have been reduced continuously through modifications of the operative procedure and through general progress in diagnosis and peri- and postoperative management. However, long-term survival following curative resection of ampullary carcinoma remains low. The reported 5-year survival rates vary from 30 to 70% [4, 24–28]. In our study, the 5-year survival equaled 40% which is equivalent to the results of a retrospective study of a large American patient population by O'Connel et al. who reported a 5-year survival of 36.8% for a total of 3292 patients, however only 1301 of whom (40%) underwent primary surgical therapy [29]. The majority of current studies looking into the long-term follow-up of ampullary cancer are conducted multicentrically and examine the long-term results for up to a maximum of ten years following resection. The information on factors that influence the long-term prognosis following the resection of ampullary carcinomas is therefore limited. It should be noted that ampullary cancer does not occur frequently overall but constitutes a relevant proportion (20–40%) of all resected tumors of the periampullary region [2–4]. One reason for this situation is the high rate of resectability at the time of diagnosis, specified in the literature as up to 80% which is significantly higher than for pancreatic head carcinoma (20%) [3, 30]. The anatomical location and the exophytic growth pattern of ampullary carcinomas, leading to early occlusion of the bile duct and therefore often-early clinical diagnosis, explain the high resectability rate and the positive results relative to the surgical radicality of the procedure [4–6]. This is underlined by the results of our study in which a total of 131 patients (92%) received R0 resection of the primary tumor with a 10-year survival of 25% and 15-year survival of 10%, respectively. In the survival analysis (log-rank), our study identified the following prognostic factors that were accompanied by a significantly reduced long-term survival: reduced general condition at the time of surgery, intraoperative administration of PRBC, POPF, tumor stage, substantial invasion depth of the tumor, lymph node stage, histological grading, resection border, vascular and lymphatic vessel invasion, and CA 19-9 levels higher than 37 U/L. There were no significant differences in regard of overall survival between patients who underwent PPPD in comparison to the classic Whipple procedure. A postpyloric resection approach therefore appears to be safe in patients with ampullary cancer.

In the multivariate analysis lymphatic vessel invasion, intraoperative administration of PRBCs, and an elevated CA 19-9 level were identified as independent risk factors for a reduced long-term survival. There is consensus in the literature for most carcinomas of the gastrointestinal tract (esophagus, stomach, and colorectum) regarding the influence of lymph node status on long-term prognosis. Aside from the results of our study, this hypothesis is substantiated by the results of Hurtuk et al., who noted the significant influence of positive lymph node involvement on long-term survival especially for ampullary and pancreatic carcinoma [31]. The 5-year survival reported in the literature is 0 to 30% when there is positive lymph node involvement and 39 to 78% in patients who lack lymph node involvement [32]. There is controversy over the extent of the lymphadenectomy. At our clinic, partial pancreaticoduodenectomy is performed with a comprehensive lymphadenectomy, along the hepatoduodenal ligament, celiac trunk, and superior mesenteric artery. However, in a study in which the results from standard lymphadenectomy and extended lymphadenectomy were compared, there were no significant differences with respect to long-term survival (56 versus 60%) [33].

Beyond radical surgical approaches, also endoscopic treatment options for ampullary tumors exist such as endoscopic resection [34], photodynamic therapy [35], and electrofulguration [36]. Endoscopic resection can provide a safe and effective treatment option for benign ampullary tumors. If an ampullary tumor appears to be benign and the biopsy samples are negative for malignancy, endoscopic papillectomy should be considered as an initial intervention. The decision on whether to perform a subsequent pancreaticoduodenectomy should then be based on the histopathological findings. However, false negative rates of 40 to 85% have been reported for endoscopic biopsies and small ampullary cancers may be missed [37]. According to the results of our study, a tumor size smaller than 2 cm even in early tumor stages does not correlate with an improved survival. A delayed treatment may therefore be fatal. An accurate differentiation between benign and malignant lesions can only be achieved by radical pancreaticoduodenectomy. Prospective randomized trials will have to evaluate a possible benefit of endoscopic resection for small ampullary malignancies, for example, in patients with an increased operative risk score.

Howe et al. also identified lymph node metastases and a positive tumor cutting margin as risk factors for reduced long-term survival [4]. However, at the same time, these authors subdivided ampullary carcinomas into two subgroups by way of detailed histopathology and found that median survival of those tumors with a histologically verified pancreatobiliary origin was significantly lower in comparison to tumors with a histologically verified intestinal origin (22 months versus 60 months) [4]. Outerbridge reported back in 1913 that ampullary cancer could exhibit different histological origins [38]. Apart from origins in the duodenal mucosa, the epithelia of the common pancreatobiliary ductal system, pancreatic duct, or bile duct are possible points of origin. In clinical practice, due to the heterogeneity of the tumor and frequent, simultaneously present preneoplastic lesions, the exact histological origin can often be not clearly differentiated. In our study, we did not further classify ampullary carcinomas based on detailed histological findings. Kimura et al. (1994), who examined 53 patients with ampullary carcinoma, were one of the first to describe histological criteria that allow for more accurate allocation to either pancreatobiliary or intestinal origin [39]. These authors also reported that ampullary cancers of pancreatobiliary origin are more frequently accompanied by lymph node involvement and had a worse prognosis than carcinomas of an intestinal origin [39]. Zhou et al. reported in 2004 the use of cytokeratin and apomycin markers to assign ampullary cancers histologically and unambiguously to one of the two subgroups [13]. However, these methods are still not a popular standard today, even though Westgaard et al. showed that the histological subtype is an essentially more relevant prognostic factor than the otherwise typical affiliation to one of the anatomical subtypes of periampullary carcinomas [40].

Tumor recurrence after resection with curative intention remains a key problem in the long-term prognosis following radical resection. The tumor recurrence rate, as reported in the literature, varies from 28 to 44%. Examples of key manifestation regions are the liver and aortocaval lymph node metastases, as well as locoregional tumor recurrence [18, 26, 41]. Postoperative adjuvant chemotherapy [42] and radiochemotherapy [43, 44] have been shown to improve survival outcomes in patients with periampullary carcinomas. In current studies, however, patients considered for postoperative adjuvant therapy often had adverse prognostic factors, such as positive lymph node involvement, higher tumor stage, or poor tumor differentiation, compared with patients who were treated with surgery alone [45]. According to the results of our study, adjuvant therapy should be recommended for patients with positive tumor cutting margins or other risk constellations, such as lymph node involvement, tumor invasion into the surrounding tissue, or poorly differentiated tumor grade. However, there is an urgent need for further studies on the influence of adjuvant therapy on the long-term prognosis after the resection of ampullary cancer.

There are several limitations to the present study. Although the clinical data were prospectively collected, the study design and analysis are retrospective and are therefore subject to an inherent selection bias. Moreover, due to the rarity of this tumor entity, in an attempt to achieve a statistically relevant patient cohort, the patients included in this study were treated over a time period of 15 years. During this time surgical techniques, peri- and postoperative management, and the role of adjuvant therapy were not consistent with recent recommendations. However, to the best of our knowledge, this study is the first to investigate the factors that influence the long-term survival in a large patient population over a period of 15 years after the surgical treatment of ampullary carcinomas. This study may therefore help to guide future practice patterns and treatment recommendations.

## 5. Conclusion

The prognosis of ampullary carcinoma is clearly better than that of other carcinomas of the periampullary region. Nevertheless, the results of our study show that factors such as an increased tumor stage, considerable invasion depth of the tumor, positive lymph node involvement, blood vessel and lymphatic invasion of the tumor, and a CA 19-9 level higher than 37 U/L are accompanied by a reduced long-term prognosis. Subsequent adjuvant therapy remains essential especially in patients with this constellation of risk factors. However, additional studies are necessary to specify the role of adjuvant therapy in improving long-term results after the resection of ampullary carcinomas.

## Figures and Tables

**Table 1:** Characteristics of the patients.

Number of patients	*n* = 143
Gender	
♂	87 (61%)
♀	56 (39%)
Median age: years (range)	64 (33–83)
Median body mass index (range)	24.8 (13.5–38.8)
Preoperative symptoms	
Jaundice	75 (52%)
Nonspecific epigastric pain	88 (62%)
10% reduction of body weight	29 (20%)
Nausea	29 (20%)
Reduced performance status	27 (19%)
Incidental finding	12 (8%)

**Table 2:** Operative and postoperative course.

Median operation time (minutes/range)	325 (182–785)
Median intraoperative blood loss (mL/range)	500 (100–3000)
Intraoperative complications	4 (3%)
Postoperative complications	34 (24%)
Wound infection	14 (10%)
Postpancreatectomy hemorrhage (PPH)	6 (4%)
Postoperative pancreatic fistula (POPF)	12 (8%)
Bile leak	2 (1%)
Delayed gastric emptying (DGE)	8 (6%)
Reoperation	10 (7%)
In-hospital mortality	5 (3.5%)

**Table 3:** Survival and prognostic factors with respect to survival-multivariate analysis.

	*P* value	Odds ratio (95% confidence interval)
No lymphatic invasion	*P* = 0.000	0.248 (0.145–0.425)
No intraoperative administration of PRBC	*P* = 0.008	0.510 (0.311–0.836)
Preoperatively elevated CA 19-9	*P* = 0.023	1.762 (1.081–2.870)
